# AHCC Activation and Selection of Human Lymphocytes via Genotypic and Phenotypic Changes to an Adherent Cell Type: A Possible Novel Mechanism of T Cell Activation

**DOI:** 10.1155/2015/508746

**Published:** 2015-12-15

**Authors:** Loretta Olamigoke, Elvedina Mansoor, Vivek Mann, Ivory Ellis, Elvis Okoro, Koji Wakame, Hajime Fuji, Anil Kulkarni, Marie Francoise Doursout, Alamelu Sundaresan

**Affiliations:** ^1^Texas Southern University, Houston, TX 77004, USA; ^2^Hokkaido School of Pharmacy, Sapporo 004-0839, Japan; ^3^Amino Up, Sapporo 004-0839, Japan; ^4^University of Texas Medical School, Houston, TX 77004, USA

## Abstract

Active Hexose Correlated Compound (AHCC) is a fermented mushroom extract and immune supplement that has been used to treat a wide range of health conditions. It helps in augmentation of the natural immune response and affects immune cell activation and outcomes. The goal of this project was to study and understand the role and mechanisms of AHCC supplementation in the prevention of immunosuppression through T cell activation. The method described here involves “*in vitro*” culturing of lymphocytes, exposing them to different concentrations of AHCC (0 *μ*g/mL, 50 *μ*g/mL, 100 *μ*g/mL, 250 *μ*g/mL, and 500 *μ*g/mL) at 0 hours. Interestingly, clumping and aggregation of the cells were seen between 24 and 72 hours of incubation. The cells lay down extracellular matrix, which become adherent, and phenotypical changes from small rounded lymphocytes to large macrophage-like, spindle shaped, elongated, fibroblast-like cells even beyond 360 hours were observed. These are probably translated from genotypic changes in the cells since the cells propagate for at least 3 to 6 generations (present observations). RNA isolated was subjected to gene array analysis. We hypothesize that cell adhesion is an activation and survival pathway in lymphocytes and this could be the mechanism of AHCC activation in human lymphocytes.

## 1. Introduction

The immune system is a dynamic network of cells tissues and organs which protects the body from infection. The immune system has beneficial and nonbeneficial aspects and fights against invading foreign materials. This can be linked to genetic makeup and thus paves a way for novel prevention and treatment measures to be generated against infectious and immune mediated diseases [1]. Being an intricate system, it comprises two major divisions that encompass various cell types which have unique roles: the innate immune system which involves a nonspecific defense mechanism and adaptive immune system which involves antigen specific immune response. A proper understanding of the different cell types that make up these systems and their mode of communication and action would give researchers an in-depth knowledge of the immune system as a whole [2]. Further in-depth knowledge on how they behave under physiological stress will also help in the study of immune augmenting agents.

We used lymphocytes as our model choice since they are responsible for the astounding specificity of the adaptive immune response. They are well populated in the human body about 2 × 10^12^ lymphocytes especially in the blood, lymph, and also the lymphoid organs such as thymus, lymph nodes, spleen, and appendix [3, 4]. Despite their abundance, their central role in adaptive immunity was not demonstrated until the late 1950s. The crucial experiments were performed in mice and rats that were heavily irradiated to kill most of their white blood cells, including lymphocytes. This treatment makes the animals unable to mount adaptive immune responses. Then, by transferring various types of cells into the animals, it was possible to determine which cells reversed the deficiency. Only lymphocytes restored the adaptive immune responses of irradiated animals, indicating that lymphocytes are required for these responses [3].

It is seen that both immune systems, innate and adaptive, work hand in hand to eliminate harmful foreign pathogens, such that an activation of the innate immune system usually at the site of infection could trigger a lymphocytic response. Unlike innate immune responses, adaptive responses are activated in peripheral lymphoid organs and provide specific and long-lasting protection against the particular pathogen that induced them.

**Scheme 1:**
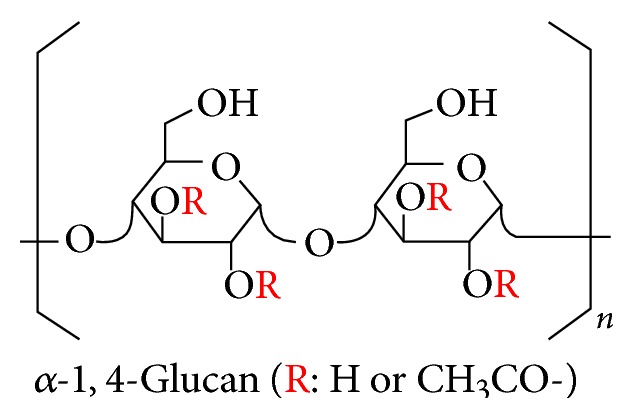
Structure of AHCC [8].

Different strategies have been looked into to try and develop a favorable approach that aids in boosting of our immune cells and so for this reason a number of substances are being synthesized, produced, extracted, and thus used for augmentation of the immune system [5]. Most of these substances are desirable since they have proven to be nontoxic to humans. AHCC is a dietary nutritional supplement/extract prepared from mycelia of the basidiomycetemushroom* Lentinula edodes* [5, 6]. It is a complex compound that contains a mixture of polysaccharides, amino acids, lipids, and minerals [7]. It is readily bioavailable and has been seen to have no adverse toxic health effect [8]. The main component of AHCC is acetylated *α*-glucan, which is relatively much easier to absorb than *β*-glucan (the main component of mushroom products) [8]. *α*-1,4-Glucan has a much lower molecular weight (5,000 Daltons) than *β*-glucan (10,000–500,000 Daltons) [7, 8]. From various experiments, which have been performed, AHCC is seen to have anticancer action, immunostimulative action, and reduction of side effects due to chemotherapy as well as defence against infection [8]. Partially acetylated alpha-glucans are known active components of AHCC as per Amino Up (see Scheme 1).

## 2. Materials and Methods

### 2.1. Materials

They include RPMI 1640 medium is purchased from Sigma-Aldrich Co. (St. Louis, MO, USA), 10% Fetal Bovine Serum (FBS), 5% Pen/Strep, and buffy coats are purchased from the Gulf Coast Regional Blood Center in Houston Texas (units accepted are those that test normal nonreactive or negative for alt, hbc, hbs, hcv, h17, hti, and sts), Hanks Balanced Salt Solution (HBBS), ficoll, and Active Hexose Correlated Compound (AHCC) are obtained from Amino Up Chemical, Japan, and Phosphate Buffer Saline (PBS), acetic acid 0.3%, trypan blue, and RNeasy mini kit are purchased from Qiagen, USA (Cat. number 74104, Inverted Microscope: Eclipse TS100 (Nikon)).

### 2.2. Complete Media Preparation

For culturing of lymphocytes isolated from normal human buffy coat, complete RPMI media has to be used. Each bottle contains 500 mL of RPMI media. This was supplemented with 10% Fetal Bovine Serum (FBS) and 5% Penicillin-Streptomycin (Pen-Strep). Complete media was prepared by taking out 50 mL of RPMI from the 500 mL bottle and adding 50 mL of FBS and 5 mL of Pen-Strep.

### 2.3. Lymphocyte Isolation

The buffy coat unit (60 mL) was diluted in 100 mL of Hanks Balanced Salt Solution (HBBS) in a T-flask for a total volume of 160 mL diluted buffy coat and thoroughly mixed. 10 mL of ficoll was added to 4 polypropylene 50 mL centrifuge tubes each and was layered with 40 mL of buffy coat at a slow steady pace preventing penetration of the ficoll layer. The tubes were then centrifuged at 2100 rpm (700 to 800 g) for 20 minutes at room temperature. (Two 50 mL tubes with 10 mL of HBBS were prepared.) Using 1 mL pipette, lymphocytes layer, formed above the ficoll, was aspirated and placed into prepared tubes. HBBS was added to top it off and centrifuged at 2100 (700–800 g) for 12 minutes at room temperature. One tube was prepared containing 10 mL of HBBS to which pelleted lymphocytes from the 2 tubes were added and centrifuged at 2100 rpm (700–800 g) for 12 minutes at room temperature. The supernatant was discarded and the resulting pellet was resuspended in 20 mL of complete RPMI. Cell counts were performed after which the AHCC experiment was set up.

### 2.4. Cell Counts

We combined 50 *μ*L of the lymphocyte cell suspension with 500 *μ*L trypan blue and 950 *μ*L of 0.3% acetic acid in an eppendorf tube and mixed well to obtain an appropriate dilution. Then, we covered a clean haemocytometer with a cover slip and using a pipettor we added, from an angle, a small amount of the stained, diluted cell suspension onto the covered haemocytometer without moving the cover slip. We allowed the cells to settle before counting.

Once we obtained the cell counts, the cell concentration was calculated using the following formula:

(1) Cell concentration=Total number of cells4×dilution factor30×104.

### 2.5. AHCC Preparation and Experimental Setup

In the manufacturing process of AHCC, it was sterilized at 121 degrees C for 45 minutes after separation of insoluble fungal parts. Then, this AHCC sample obtained from Amino Up Chemical, Japan, is freshly prepared in sterile complete media (RPMI1640) at a 5 mg/mL stock concentration. Total volume of this sample prepared varies as per experimental need. Cells were resuspended at 1 *∗* 10^6^ cells/mL in 10 mL tissue culture flasks in triplicate. Each flask corresponds to the different experimental concentrations and the corresponding volumes of AHCC were added to achieve the desired concentrations: 0 *μ*g/mL (control), 50 *μ*g/mL, 100 *μ*g/mL, 250 *μ*g/mL, and 500 *μ*g/mL. The flasks containing the cell cultures were incubated at optimal conditions 37°C and 5% CO_2_. Daily observations were recorded and microscopic images taken over several days.

### 2.6. MTT Assay

The cells were isolated, counted, and plated to 96-well plates on the same day as blood collection. 97 *μ*L of RPMI1640 culture medium was added to 21 wells of a 96-well plate. A final volume of 100 *μ*L/well was achieved by adding 3 *μ*L of cell suspension (containing 1 × 10^5^ cells) into each well. To the wells designated for the different AHCC, 0 *μ*g/mL (control), 50 *μ*g/mL, 100 *μ*g/mL, 250 *μ*g/mL, and 500 *μ*g/mL concentrations corresponding volumes of AHCC were added to achieve the desired concentrations. The cells were incubated at 37°C for 48 hours and then dye solution was added to each well (15 *μ*L/well) and incubated for 4 hours in a humidified, 5% CO_2_ atmosphere. After 4 hours of incubation, the solubilization solution/stop mix was added (100 *μ*L/well) to stop the cellular conversion of the MTT dye and was again incubated for 1 hour. Finally, cell proliferation was measured using Tecan 96-well plate reader and absorbance at 570 nm wavelength was recorded* (CellTiter 96 Non-Radioactive Cell Proliferation Assay, Promega Corporation, Madison, WI, USA)*.

### 2.7. RNA Isolation

RNA was isolated according to the protocol obtained from the RNeasy mini kit purchased from Qiagen.

### 2.8. RNA Analysis by Illumina

Three hundred nanograms of total RNA was amplified and purified using Illumina TotalPrep RNA Amplification Kit (Ambion, Cat.# IL1791) following kit instructions. Briefly, first strand cDNA was synthesized by incubating RNA with T7 oligo (dT) primer and reverse transcriptase mix at 42°C for 2 hours. RNase H and DNA polymerase master mix were immediately added to the reaction mix following reverse transcription and were incubated for 2 hours at 16°C to synthesize second strand cDNA. RNA, primers, enzymes, and salts that would inhibit* in vitro* transcription were removed through cDNA filter cartridges (part of the amplification kit).* In vitro* transcription was performed and biotinylated cRNA was synthesized by 14-hour amplification with dNTP mix containing biotin-dUTP and T7 RNA polymerase. Amplified cRNA was subsequently purified and concentration was measured by NanoDrop ND-1000 Spectrophotometer (NanoDrop Technologies, DE). An aliquot of 750 nanograms of amplified products was loaded onto Illumina Sentrix Beadchip Array HumanHT12_V4 arrays, hybridized at 58°C in an Illumina Hybridization Oven (Illumina, Cat.# 198361) for 17 hours, washed, and incubated with streptavidin-Cy3 to detect biotin-labeled cRNA on the arrays. Arrays were dried and scanned with BeadArray Reader (Illumina, CA). Data were analyzed using GenomeStudio software (Illumina, CA). Clustering and pathway analysis were performed with GenomeStudio and Ingenuity Pathway Analysis (Ingenuity Systems, Inc.) software, respectively.

### 2.9. Statistical Analysis

Standard error was calculated based on the mean and the standard deviation and the standard error bars were included in the data. A “*P*” value less than 0.05 was considered statistically significant.

## 3. Results and Discussion

### 3.1. Phenotypic Observations of the Cells

**Figure 1:**
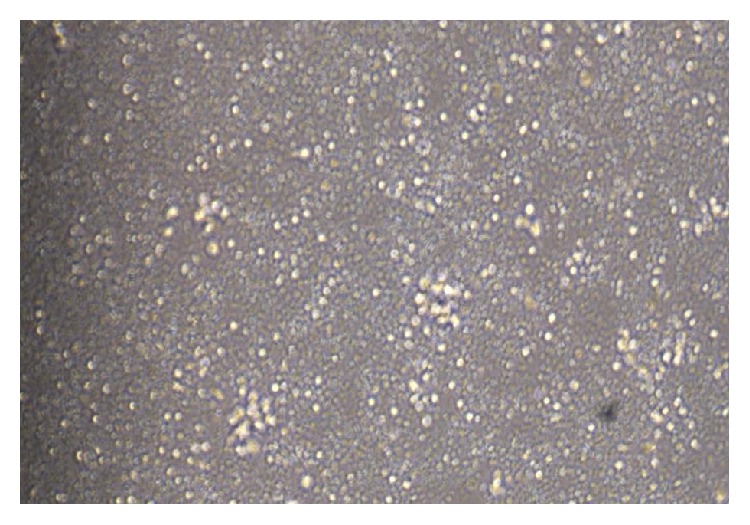
Control-0 *μ*g/mL treated lymphocytes, 96 hours.

**Figure 2:**
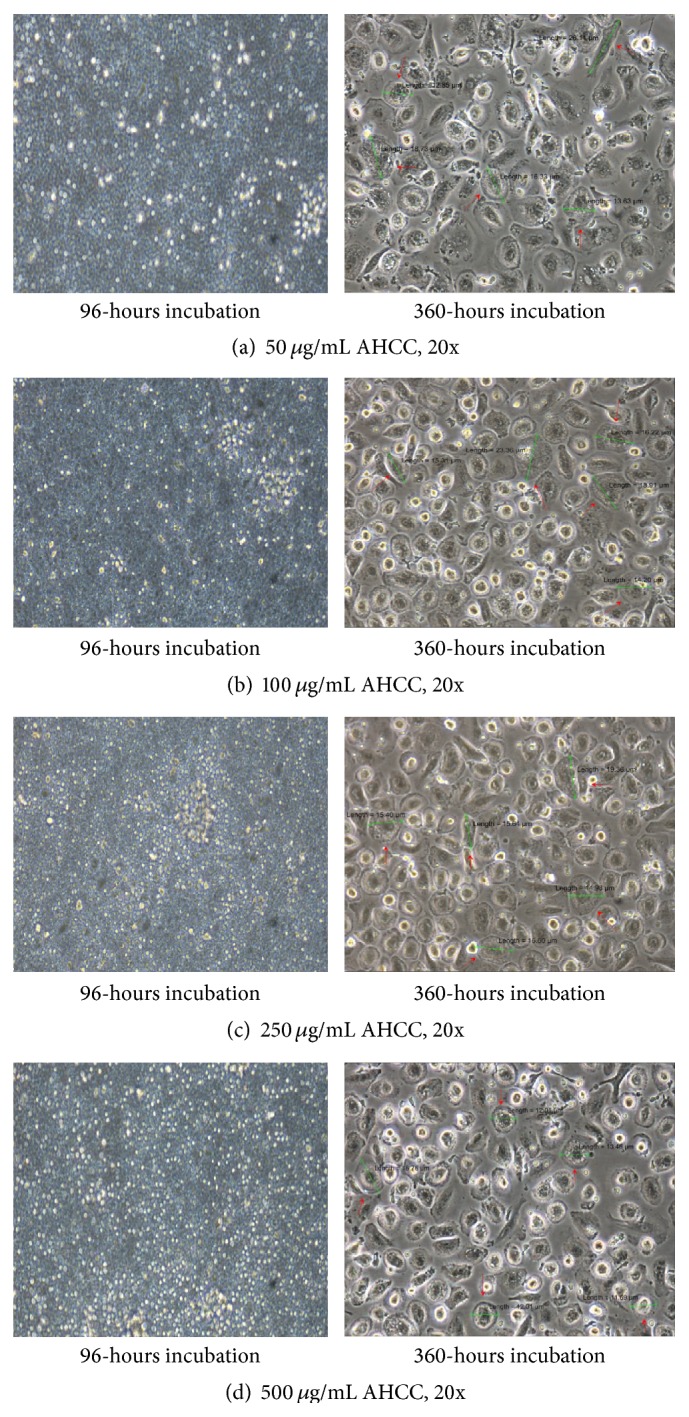
Representative image of different concentrations of AHCC treated lymphocytes: (a) 50 *μ*g/mL AHCC 20x, (b) 100 *μ*g/mL AHCC 20x, (c) 250 *μ*g/mL AHCC, and (d) 500 *μ*g/mL AHCC. Note: the morphological changes of lymphocytes from 96 hours to 360 hours posttreatment with AHCC. Cells become adherent and phenotypical changes were observed even at low doses such as 50 *μ*g/mL (a) that is macrophage-like, spindle shaped, elongated, and fibroblast-like up until 360 hours and beyond. Similar changes were observed in the other doses: 100 *μ*g/mL to 500 *μ*g/mL (b, c, d).

**Figure 3:**
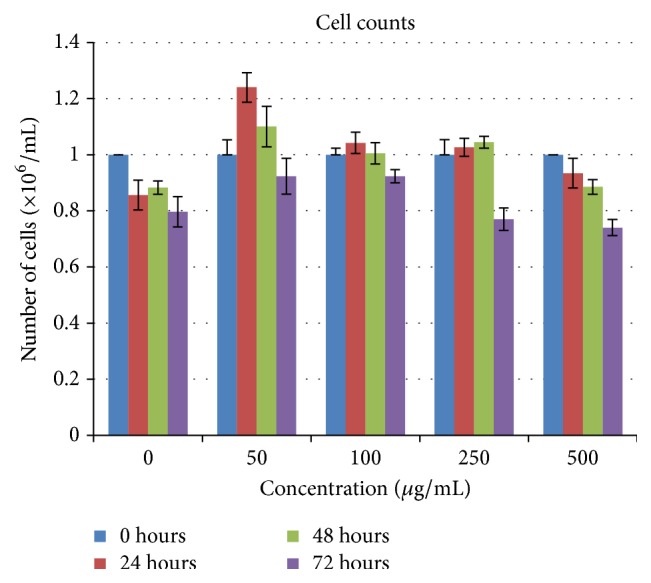
Cell counts until 72 hours: overall increase in the cell count is observed 24 hours after culture (except for the control flask) which later decreases over 48 and 72 hours as the cells begin to aggregate, clump, and adhere to the bottom of the flask. The most significant increase was seen in 50 *μ*g/mL from “0” hours to “24” hours compared to the other treated flasks. (Note that lymphocytes are resting cells which have been induced to multiply.)

**Figure 4:**
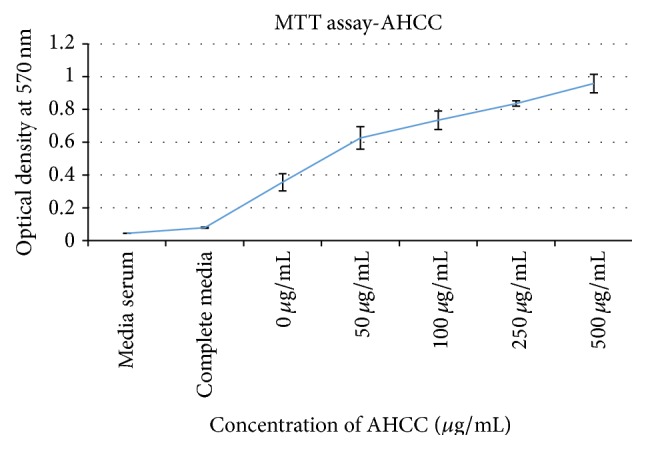
Cell proliferation data for AHCC treated lymphocytes. 1 × 10^5^ cells were seeded into the desired well of the 96-well plate. These cells were then treated with different concentrations of the nutritional supplement AHCC at “0” hours. At 48 hours after culture, the cells were treated with MTT reagent according to the protocol. Absorbance was measured at 570 nm.

**Figure 5:**
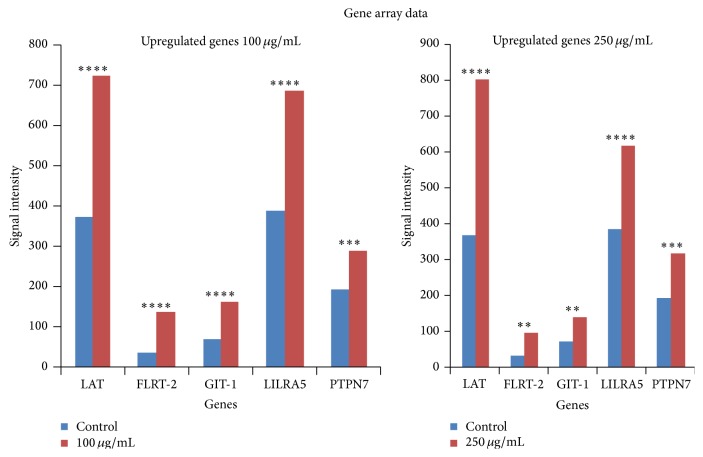
Graphical representation of genes responsible for cell proliferation, adhesion, differentiation, receptor signaling, and signal transduction leading to activation of the immune system thus evoking an immune response. It shows upregulated genes (100 *μ*g/mL and 250 *μ*g/mL concentrations); their corresponding signal intensity after a gene array analysis was performed. *P* < 0.05 was considered statistically significant. “*∗∗*” means *P* < 0.01, “*∗∗∗*” means *P* < 0.001, and “*∗∗∗∗*” means *P* < 0.0001.

**Figure 6:**
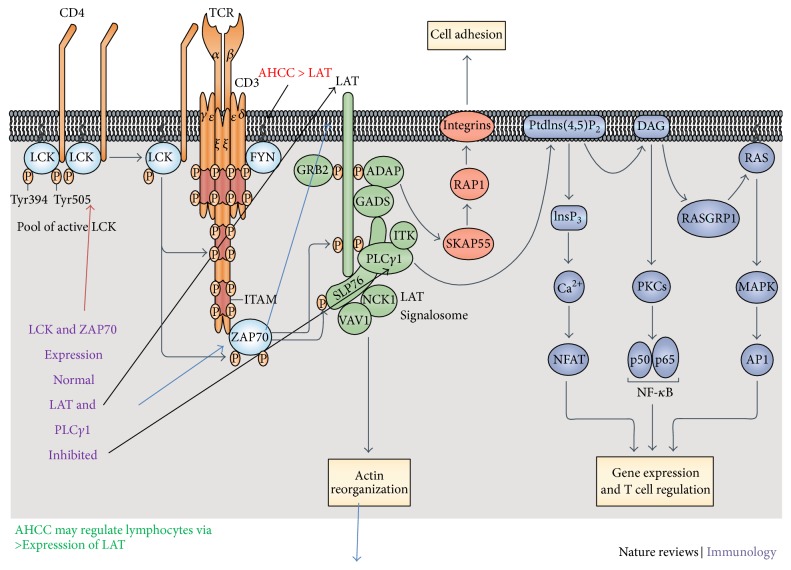
T cell receptor (TCR) signal transduction is initiated by the recognition of cognate peptide-MHC molecules. The first molecule to be recruited to the TCR-CD3 complex is the SRC family kinase (SFK) member LCK, which phosphorylates immunoreceptor tyrosine-based activation motifs (ITAMs) of the CD3*γ* chain, CD3*δ* chain, CD3*ε* chains, and the *ζ*-chains.

Control-0 *μ*g/mL treated lymphocytes, as shown in Figure 1, live only until 96 hours and die thereafter. Interestingly in AHCC treated cells, after a time lag, clumping and aggregation of the cells were seen between 24 and about 72 hours of incubation. The cells then become* adherent* and* phenotypical changes* were observed even at low doses such as 50 *μ*g/mL (Figure 2(a)), that is, macrophage-like, spindle shaped, elongated, and fibroblast-like up until 360 hours and beyond. Similar changes were observed in the other doses: 100 *μ*g/mL to 500 *μ*g/mL (Figures 2(b), 2(c), and 2(d)). Cell proliferation also increased (cell count assays). The cells were not subjected to stress as the media was changed at various intervals, so stress due to nutrient depletion was eliminated. These are probably translated from genotypic changes in the cells since the cells propagate for at least three to six generations (present observations). Cell count data is shown for up to 72 hours since after that cells become adherent and cannot be counted. Cell viability was high throughout, indicating that AHCC is able to boost cell health (Figure 3). Cell proliferation was further demonstrated via the cell metabolism and proliferation MTT assay (Figure 4). This further elaborated that there was a dose response dependent increase of resting lymphocyte proliferation in response to AHCC treatment. The AHCC treated adherent phenotypic cells can be trypsinized for two generations after which they visibly lay down a lot of extracellular matrixes (ECM). These genotypic changes possibly involve signal transduction of cell adhesion molecules and adhesive cytokines and growth factors. Cell adhesion is an activation and survival pathway in lymphocytes and we hypothesize that this could be the mechanism of AHCC activation in human lymphocytes. From the gene array analysis it was seen that certain genes responsible for T cell proliferation and differentiation as well as cell adhesion were significantly upregulated (Figure 5). Of great importance is the LAT gene (linker for activation of T cells) which is required for TCR (T cell antigen receptor) and pre-TCR-mediated signaling in both developing and mature T cells. The possible mode of LAT action in lymphocyte activation in response to AHCC in our experiments is illustrated in Figure 6. In our experiments, AHCC possibly induces upregulation of LAT and promotes cell adhesion to activate resting lymphocytes.

Results are described as representative of five experiments with the following concentrations: 0 *μ*g/mL, 50 *μ*g/mL, 100 *μ*g/mL, 250 *μ*g/mL, and 500 *μ*g/mL.

The control flask, after a period of about 96 hours, dies off as the cells are not treated with AHCC and so there is no further stimulation of growth/proliferation. From 50 *μ*g/mL to 500 *μ*g/mL, a pictorial representation is given showing 96 hours and 360 hours of incubation (Figures 2(a)–2(d)). The differences in the phenotypic appearance of the cells are distinct; these cells being activated by AHCC adhere, differentiate, and also vary in sizes ranging from about 11 *μ*m to about 26 *μ*m in diameter. It was also observed that these cells once in contact with each other do not exhibit a contact inhibition, but they begin to merge resulting in a single cell and also continue to grow within the plate. After reaching a confluent stage, the cells were trypsinized and passaged and, after a time lag, the exact same phenomenon was observed. It should be noted that AHCC was only added once, at the start of the experiment, and supplemented with complete media when needed even after cell adhesion had occurred, to avoid nutrient deprivation. The exact mechanism of how AHCC acts on and activates these cells causing them to proliferate, adhere, and differentiate is unknown.

### 3.2. Cell Counts

After isolation of these lymphocytes, they were resuspended at a concentration of 1 × 10^6^ cells/mL for a total of 10 mL. This was done for all concentrations. A cell count gives an idea of cell proliferation, that is, live versus dead cells. In order to assess if AHCC induced cell proliferation after treatment, cells were counted and we found that after 24 hours an increase was observed in the treated flasks as opposed to the control flask. From 48 to 72 hours there was an observable decrease in the cell count (Figure 3); the reason for this is that, at this period of time, the cells began to aggregate and so a true cell count could not be achieved. For this reason, cell counts were only carried out for 3 days from experimental setup.

### 3.3. MTT Assay

The CellTiter 96 Non-Radioactive Cell Proliferation Assay is a collection of qualified reagents that provide a rapid and convenient method of determining viable cell number in proliferation, cytotoxicity [9, 10], cell attachment [11, 12], chemotaxis [13], and apoptosis [14] assays. The MTT assay is a colorimetric assay for assessing cell metabolic activity. NAD(P)H-dependent cellular oxidoreductase enzymes may, under defined conditions, reflect the number of viable cells present. These enzymes are capable of reducing the tetrazolium dye MTT 3-(4,5-dimethylthiazol-2-yl)-2,5-diphenyltetrazolium bromide to its insoluble formazan product, which has a purple color; a solubilization solution is added to dissolve the insoluble purple formazan product into a colored solution that is easily detected using a 96-well plate reader. The absorbance of this colored solution can be quantified by measuring at a certain wavelength (usually between 500 and 600 nm) by a spectrophotometer [15].

The CellTiter 96 Assay has several advantages over conventional cell number or 3[H]thymidine incorporation assays. There is a minimum amount of labor involved in doing the CellTiter 96 Assay. The assay is done entirely in a 96-well plate with no steps that require washing the cells or the removal of solution from the wells. The assay can be used for both anchorage-dependent or suspension cells with no change in the protocol. The assay plates are read using a 96-well plate reader, making it easy to computerize data collection, calculations, and report generation [16].

MTT assay gives an idea of cell metabolism and is widely used to measure metabolic activity; thus, it is considered a real proliferation assay. Our results show a significant increase from control (0 *μ*g/mL) to 500 *μ*g/mL.

### 3.4. Gene Array Data

This phenomenon of adhesion has previously not been seen in previous experiments conducted with lymphocytes treated with other agents. In order to delineate the cause of these genotypic and phenotypic effects, RNA isolated from harvested cells is subjected to a gene array analysis to get a bigger picture. This is then narrowed down to pathways that get turned on as a result of AHCC treatment, as the phenotypic effects are seen after a genotypic change has occurred.

In order to specifically characterize the unique phenotypic and proliferation phenomena seen with AHCC treatment in resting lymphocytes compared to control, we analyzed cells harvested and subjected them to the Illumina platform.

From the gene array data that was obtained (Figure 5), a group of significant genes LAT, FLRT-2, GIT-1, LILRA5, and PTPN7 responsible for cell proliferation, cell adhesion and/or receptor signaling, triggering of innate immune responses, differentiation, and regulation of T-lymphocytes as well as signal transduction were identified. Of primary importance is the LAT gene. The gene array data revealed possible direct activation of the lymphocytes via direct engagement of the T cell receptor complex. The linker for activated T cells (LAT), which is the primary activator after TCR engagement in AHCC treated lymphocytes, increased by 2.7-fold compared to control even at 360 hours posttreatment. This is high for resting cells. As an adaptor protein, the function of LAT in TCR signaling centers upon its tyrosine phosphorylation and subsequent recruitment of other signaling proteins. Upon TCR engagement, phosphorylation of LAT allows it to interact with several SH2 domain-containing proteins, such as Grb2, Gads, and PLC-*γ*1. We have already seen inhibition of LAT and PLC-*γ*1 in lymphocytes in microgravity (an immune suppressive scenario) related to reduced T cell activation. Hence, AHCC might be able to directly activate LAT and thus might be a possible future countermeasure candidate to restore T cell activation in immunosuppressive scenarios. It is already in human use as an immune augmentation supplement in conjunction with cancer chemotherapy. AHCC thus might be a possible future countermeasure candidate to restore T cell activation in immunosuppressive scenarios on earth and in space.

Phosphorylation of the ITAMs enables the recruitment of ZAP70 (*ζ*-chain associated protein kinase of 70 kDa), its phosphorylation by LCK, and its activation. Activated ZAP70 phosphorylates four key tyrosine residues on linker for activation of T cells (LAT), which recruits numerous signaling molecules to form a multiprotein complex, termed the LAT signalosome. Important molecules that constitute this complex include phospholipase C*γ*1 (PLC*γ*1), growth factor receptor-bound protein 2 (GRB2), GRB2-related adaptor protein GADS, SLP76 (SH2 domain-containing leukocyte protein of 76 kDa), adhesion- and degranulation-promoting adaptor protein (ADAP), interleukin-2-inducible T cell kinase (ITK), NCK1, and VAV1. The LAT signalosome propagates signal branching to three major signaling pathways, the Ca^2+^, the mitogen-activated protein kinase (MAPK) kinase, and the nuclear factor-*κ*B (NF-*κ*B) signaling pathways, leading to the mobilization of transcription factors that are critical for gene expression and essential for T cell growth and differentiation. Signals initiated from the TCR also result in actin reorganization and the activation of integrins by inside-out signaling. Note the following abbreviations: AP1, activator protein 1; DAG, diacylglycerol; InsP_3_, inositol-1,4,5-trisphosphate; NFAT, nuclear factor of activated T cells; PKC, protein kinase C; PtdIns(4,5)P_2_, phosphatidylinositol-4,5-bisphosphate; RASGRP1, RAS guanyl-releasing protein 1; SKAP55, SRC kinase-associated phosphoprotein of 55 kDa [17]. Our key culprits implicated thus far from our experiments are LAT, PLC-*γ*1, and ZAP 70. AHCC thus might modulate LAT and promote cell adhesion.

FLRT-2 is a gene that encodes a member of the fibronectin leucine-rich transmembrane protein (FLRT) family. FLRT family members may function in cell adhesion and/or receptor signaling. Their protein structures resemble small leucine-rich proteoglycans found in the extracellular matrix (provided by Ref. Seq., Jul. 2008).

GIT-1 (G Protein-Coupled Receptor Kinase Interacting ArfGAP 1) is a protein coding gene. GTPase-activating protein for the ADP ribosylation factor family, which may serve as a scaffold to bring together molecules to form signaling modules controlling vesicle trafficking, adhesion, and cytoskeletal organization, increases the speed of cell migration, as well as the size and rate of formation of protrusions. Among its related pathways are regulations of actin cytoskeleton. It localizes synaptically especially in dendritic cells [18]. If synaptic localization is disturbed, dendritic cells lose their morphology. It is responsible for differentiation and preservation of dendritic cell morphology and it interacts with PLC*γ*1.

LILRA5 leukocyte immunoglobulin-like receptor, subfamily A, member 5: the protein encoded by this gene is a member of the leukocyte immunoglobulin-like receptor (LIR) family. LIR family members are known to have activating and inhibitory functions in leukocytes. Crosslink of this receptor protein on the surface of monocytes has been shown to induce calcium flux and secretion of several proinflammatory cytokines, which suggests the roles of this protein in triggering innate immune responses (provided by Ref. Seq., Jul. 2008).

PTPN7 protein tyrosine phosphatase, nonreceptor type 7: the protein encoded by this gene is a member of the protein tyrosine phosphatase (PTP) family. PTPs are known to be signaling molecules that regulate a variety of cellular processes including cell growth, differentiation, mitotic cycle, and oncogenic transformation. This gene is preferentially expressed in a variety of hematopoietic cells and is an early response gene in lymphokine stimulated cells. The noncatalytic N-terminus of this PTP can interact with MAP kinases and suppress the MAP kinase activities. This PTP was shown to be involved in the regulation of T cell antigen receptor (TCR) signaling, which was thought to function through dephosphorylating the molecules related to MAP kinase pathway. Multiple alternatively spliced transcript variants have been found for this gene (provided by Ref. Seq., Dec. 2010).

## 4. Conclusion

From the above concrete observations, it can be hypothesized that AHCC is promoting proliferation and differentiation of leukocytes (multipotent stem cells), as it is seen that the genes responsible for these such as LAT, FLRT2, GIT-1, and so forth, as seen in Figure 5, are upregulated and highly significant (*P* < 0.0005). Gene array and adhesion molecule experiments are ongoing. Cell adhesion is an activation and survival pathway in lymphocytes and we hypothesize that this could be the mechanism of AHCC activation in human lymphocytes. This is a unique phenomenon and has previously not been observed before in our laboratory when we used other agents such as mitogens, transferrin, and nucleotides for rescue of lymphocyte activation and locomotion. We have conducted these studies up to date in 8 normal donors and have found this phenomenon to be donor independent. The study of the adhesion molecules and the ECM could provide valuable insight into the mode of action of AHCC in augmenting the immune system, which has been observed in human subjects. AHCC has immunomodulatory effects via T cell activation, which could be useful for further studies in immunosuppression in microgravity and stressed physiological environments. Physical stressors include microgravity, radiation, malnutrition, and microbial contamination and atmospheric pollutants due to recycled air and water [19, 20]. Microgravity is one of the most detrimental stressors for space travelers and aggravates bone structure and composition, immune function, and the psychoneuroendocrine system and induces muscle atrophy. Immune suppression in microgravity has been documented for many years. With human exploration and long-term space travel, the immune system of the astronaut has to be optimally maintained. Individual risks to organs, such as the heart, bone, muscle, and the immune system, occur in microgravity.

Spaceflight causes various changes in the immune system, including decreases in T cell proliferation, cell-mediated activity, natural killer (NK) cell activity, macrophage function, and responses of bone marrow cells to colony stimulating factors and alteration in cytokine production [19, 20]. However, because of the limited sample number, different mission durations, and different experimental conditions, spaceflight-induced immune changes have not been fully elucidated and ground-based studies have also been required. AHCC (Active Hexose Correlated Compound, Amino Up, Japan) might potentially play a core nutritional metabolic role in regulating physiological stress. Since our lab studies lymphocyte activation in immunosuppressive scenarios such as microgravity, the next step is to expose lymphocytes to modeled microgravity (mmg) using the NASA rotating wall vessel, and unit earth gravity or 1 g, in the presence and absence of the same concentrations of AHCC to evidence if LAT is upregulated in mmg AHCC treated lymphocytes compared to controls grown in mmg alone.

## References

[1] http://www.niaid.nih.gov/topics/immunesystem/Pages/default.aspx.

[2] http://www.niaid.nih.gov/topics/immuneSystem/Pages/overview.aspx.

[3] Alberts B., Johnson A., Lewis J. (2002). Lymphocytes and the cellular basis of adaptive immunity. *Molecular Biology of the Cell*.

[4] http://bionumbers.hms.harvard.edu/bionumber.aspx?&id=103587&ver=0.

[5] Yin Z., Fujii H., Walshe T. (2010). Effects of active hexose correlated compound on frequency of CD4^+^ and CD8^+^ T cells producing interferon-*γ* and/or tumor necrosis factor—*α* in healthy adults. *Human Immunology*.

[6] Roman B. E., Beli E., Duriancik D. M., Gardner E. M. (2013). Short-term supplementation with active hexose correlated compound improves the antibody response to influenza B vaccine. *Nutrition Research*.

[7] Nogusa S., Gerbino J., Ritz B. W. (2009). Low-dose supplementation with active hexose correlated compound improves the immune response to acute influenza infection in C57BL/6 mice. *Nutrition Research*.

[8] http://www.aminoup.co.jp/e/products/AHCC/.

[9] Campling B. G., Pym J., Galbraith P. R., Cole S. P. C. (1988). Use of the mtt assay for rapid determination of chemosensitivity of human leukemic blast cells. *Leukemia Research*.

[10] Jover R., Ponsoda X., Castell J. V., Gómez-Lechón M. J. (1994). Acute cytotoxicity of ten chemicals in human and rat cultured hepatocytes and in cell lines: correlation between in vitro data and human lethal concentrations. *Toxicology In Vitro*.

[11] Klemke R. L., Yebra M., Bayna E. M., Cheresh D. A. (1994). Receptor tyrosine kinase signaling required for integrin alpha v beta 5-directed cell motility but not adhesion on vitronectin. *The Journal of Cell Biology*.

[12] Prieto A. L., Edelman G. M., Crossin K. L. (1993). Multiple integrins mediate cell attachment to cytotactin/tenascin. *Proceedings of the National Academy of Sciences of the United States of America*.

[13] Shi Y., Kornovski B. S., Savani R., Turley E. A. (1993). A rapid, multiwell colorimetric assay for chemotaxis. *Journal of Immunological Methods*.

[14] Wong G. H. W., Goeddel D. V. (1994). Fas antigen and p55 TNF receptor signal apoptosis through distinct pathways. *The Journal of Immunology*.

[15] https://en.wikipedia.org/wiki/MTT_assay.

[16] http://www.promega.com/~/media/files/resources/protocols/technical%20bulletins/0/celltiter%2096%20non-radioactive%20cell%20proliferation%20assay%20protocol.pdf.

[17] Brownlie R. J., Zamoyska R. (2013). T cell receptor signalling networks: branched, diversified and bounded. *Nature Reviews Immunology*.

[18] http://www.genecards.org/cgi-bin/carddisp.pl?gene=GIT1&keywords=GIT1.

[19] Aponte V. M., Finch D. S., Klaus D. M. (2006). Considerations for non-invasive in-flight monitoring of astronaut immune status with potential use of MEMS and NEMS devices. *Life Sciences*.

[20] Borchers A. T., Keen C. L., Gershwin M. E. (2002). Microgravity and immune responsiveness: implications for space travel. *Nutrition*.

